# Bridging molecular to cellular scales for models of membrane receptor signaling

**DOI:** 10.1101/2024.12.04.626844

**Published:** 2024-12-05

**Authors:** Kelvin J. Peterson, Boris M. Slepchenko, Leslie M. Loew

**Affiliations:** R. D. Berlin Center for Cell Analysis and Modeling, University of Connecticut School of Medicine, Farmington, CT USA

## Abstract

Biochemical interactions at membranes are the starting points for cell signaling networks. But bimolecular reaction kinetics are difficult to experimentally measure on 2-dimensional membranes and are usually measured in volumetric *in vitro* assays. Membrane tethering produces confinement and steric effects that will significantly impact binding rates in ways that are not readily estimated from volumetric measurements. Also, there are situations when 2D reactions do not conform to simple kinetics. Here we show how highly coarse-grained molecular simulations using the SpringSaLaD software can be used to estimate membrane- tethered rate constants from experimentally determined volumetric kinetics. The approach is validated using an analytical solution for dimerization of binding sites anchored via stiff linkers. This approach can provide 2-dimensional bimolecular rate constants to parameterize cell-scale models of receptor-mediated signaling. We explore how factors such as molecular reach, steric effects, disordered domains, local concentration and diffusion affect the kinetics of binding. We find that for reaction-limited cases, the key determinant in converting 3D to 2D rate constant is the distance of the binding sites from the membrane. On the other hand, the mass action rate law may no longer be obeyed for diffusion-limited reaction on surfaces; the simulations reveal when this situation pertains. We then apply our approach to epidermal growth factor receptor (EGFR) mediated activation of the membrane-bound small GTPase Ras. The analysis reveals how prior binding of Ras to the allosteric site of SOS, a guanine nucleotide exchange factor (GEF) that is recruited to EGFR, significantly accelerates its catalytic activity.

## Introduction.

The cell membrane responds to and integrates electrical, mechanical and chemical signals from the extracellular environment. For chemical signals, the initial step is binding of a ligand to an external binding site on a membrane receptor protein. This triggers a chain of events that typically involves a change of state of the cytoplasmic receptor domain and subsequent recruitment of adapter proteins, enzymes and/or cytoskeletal regulators to further evoke a cell biological response. Mathematical modeling of signaling pathways is a powerful tool to systematically organize the experimental knowledge we have about these complex systems and then develop predictions, through simulations, to inspire new experiments (1, 2).

A challenge in developing cell signaling models is the acquisition of the appropriate experimentally-grounded input parameters. Often, kinetic data is available from *in vitro* biochemistry and this has served the mature field of metabolic modeling very well. However, rate parameters are less available and more difficult to measure for signaling pathways and networks. Among the key challenges is that many of the essential steps are associated with the plasma membrane, where multiple molecules are recruited before a messenger ultimately diffuses to an intracellular target (e.g. the nucleus). It is experimentally difficult to measure reaction rates on membranes, so available data is commonly derived from volumetric measurements. While such quantitative data is useful, it can be challenging to translate rate parameters derived from 3D solution to the biophysically very different environment of a 2D membrane.

Indeed, the biophysics of membrane associated reactions has a long scientific history. An early focus of investigation, initiated with a classic paper by Adam and Delbruck (3), was the difference between 2D and 3D diffusion limited reactions; they argued that the 2-step process of absorbing a cytosolic molecule to the membrane and subsequent 2D search for an enzyme or binding partner might offer a kinetic advantage over a fully 3D search. However, this has been disputed using subsequent experimentally determined realistic parameters and biological scenarios (4). With regard to cell signaling, theoretical analyses of reactions at membranes have been extended and elaborated to consider both diffusion limited and reaction limited bimolecular kinetics(1, 5).

These earlier pioneering studies treated membrane reactions as strictly two dimensional surface events. However, most biological membrane associated reactions actually occur in the immediately adjacent cytosol, with interacting sites tethered to the membrane through lipid or protein anchors. A well-known feature of anchoring bimolecular reactions to a membrane is the effect of locally increased effective concentration: compared to the same reaction by the same number of molecules within the cell volume, anchoring the reaction to the membrane generally increases concentration by confining the reaction volume to a thin layer above the membrane (6). The thickness of that layer is often parametrized as *h*, sometimes called the “confinement length” (7, 8). Roughly, *h* is related to the distance the binding sites can sample above the membrane surface. The smaller *h*, the greater the effective concentrations of binding sites and the faster the membrane associated bimolecular kinetics. In principle, *h*, can be determined by analyzing detailed molecular dynamics simulations on the flexibility and motions of binding domains tethered to the membrane (8, 9). Recently, the concept of *molecular reach* was introduced as a more general framework for assessing how molecular structure can influence the steady state phosphorylated fraction of a membrane bound substrate interacting with a tethered kinase (10). The *reach* is defined as the distance of the kinase site from the membrane anchor and is directly related to *h* when the anchor diffuses freely in the membrane. However, when lateral diffusion is restricted (e.g. within large signaling clusters such as the immune synapse), binding sites with longer *reach* may have an advantage in being able to find more binding partners; for such diffusion-limited scenarios, this can outweigh the decrease in local concentration associated with increased *h* (10).

Thus, it is clear from these foundational studies that the membrane environment and the structural features of interacting membrane-bound molecules need to be considered in converting a measured 3D on-rate to a 2D on-rate suitable for cell-scale continuum models based on ordinary or partial differential equations (ODEs or PDEs). In this work, we show how this can be done using simulations from SpringSaLaD (11) to derive 2D rate constants. SpringSaLaD uses a series of variously sized spherical sites linked together with stiff springs to coarsely model the key structural features of macromolecules such as flexibility, excluded volume and binding site localization. Each sphere within the molecule is given its own diffusion coefficient and Brownian diffusion is simulated via a Langevin dynamics algorithm. The molecule can be tethered to a surface, representing a membrane, via a specialized anchoring sphere that can be given a lateral diffusion coefficient; the rest of the molecule, including the spheres designated as binding sites, are free to explore the volume above the membrane within their reach. Naturally therefore (and particularly advantageous for the purpose of this work), volumetric rate constants are assigned to bimolecular rate expressions even for membrane-bound molecules. For example, this feature was used to show how multivalent clustering is enhanced when one of the interacting molecules is tethered to a membrane (12).

To determine 2D rate constants, we fit the stochastic kinetics simulated with SpringSaLaD to a deterministic mass-action rate law based on the corresponding surface densities of the binding partners. Using idealized structures, we validate this procedure against analytical solutions. We explore how molecular structural features, membrane density and lateral diffusion affect the kinetics. Then, as a biologically relevant example, we apply this approach to recruitment of multivalent binding partners to the epidermal growth factor receptor (EGFR), including the binding of SOS, the G-protein exchange factor (GEF) for Ras. A better understanding of the cooperativity of SOS activation of Ras emerges from this analysis.

## Results

### Volume vs. surface binding kinetics for a simple dimerization reaction.

For bimolecular reactions, SpringSaLaD determines the microscopic probability of 2 binding sites forming a bond as they diffuse within a reaction radius that is slightly larger than the sum of their physical radii. The input to the algorithm is simply the macroscopic volumetric on rate constant ($k_{\mathrm{on}}^{\left(vol\right)}$, units of μM^−1^s^−1^) and the diffusion coefficient of the individual spheres. Full details on the derivation of the reaction probability and a thorough validation of its accuracy can be found in the original paper describing SpringSaLaD (11). Importantly for the purposes of this work, the rate of binding for sites that happen to be tethered to a membrane are still treated as volumetric, because the spherical sites are located in the volume compartment even while they are constrained with links to the 2D membrane surface.

**Figure 1.**
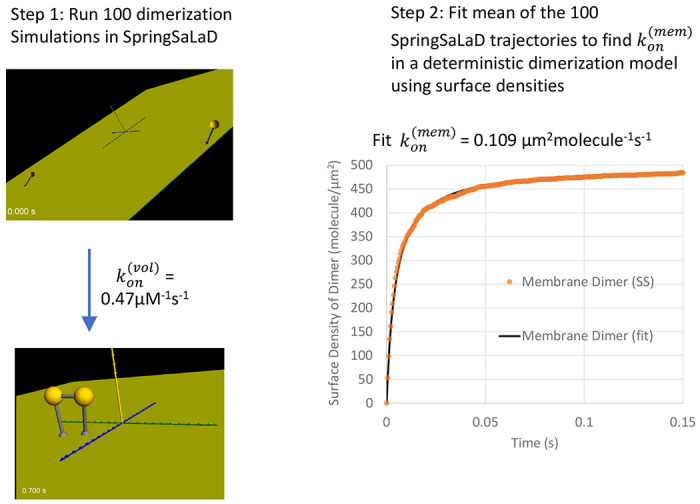
Workflow for finding membrane bimolecular binding rate constants ($k_{on}^{\left(mem\right)}$) in terms of surface densities. Step 1 is to run 100 SpringSaLaD trajectories with the coarse grained model of the binding partners. Illustrated are a pair of simple monomers (top) consisting of 2nm diameter spheres (yellow) tethered to a 1nm membrane anchor sphere (gray) with a 5nm link; both the anchor and binding spheres are assigned the diffusion coefficient of 1 μm^2^/s. At the bottom, the product dimer is depicted. Step 2 consists of fitting the average of 100 outputs from stochastic SpringSaLaD (SS) simulations to a deterministic (ODE) non-spatial model of surface-bound dimerization.

Figure 1 illustrates this for a simple dimerization reaction where the 2 yellow binding sites are tethered to the membrane anchor (gray sphere) by a 5nm link; in these simulations both the anchor and tether sites are given identical diffusion coefficients of 1μm^2^/s. We ran 100 SpringSaLaD simulations each with 40 dimerizing molecules (Fig. 1 only shows 2 for clarity). The mean trajectories for these 100 runs are then fit to a deterministic mass action membrane binding model in terms of surface densities using either COPASI or Virtual Cell (although an analytical solution can be also fit for simple dimerization) (Step 2 in Fig. 1). The output binding rate constant, $k_{on}^{\left(mem\right)}$ in units of μm^2^molecule^−1^s^−1^, can then be used to parameterize larger deterministic or stochastic models with molecule numbers (>1000) or timescales (>10 s) that would be too large for even highly coarse-grained molecular simulators like SpringSaLaD. Also, we emphasize that binding rates are typically determined experimentally using in vitro volumetric measurements; that SpringSaLaD uses volumetric rate constants as its inputs makes the procedure in Fig. 1 especially appropriate and convenient. The results in Fig. 1 show that a 2nm diameter binding site with an on rate for dimerization of 0.47 μM^−1^s^−1^ and tethered to a membrane surface through a 5nm link, can be modeled as a 2D surface reaction with an on rate, $k_{on}^{\left(mem\right)}$, of 0.109 μm^2^molecule^−1^s^−1^. Using idealized models, we now explore how various structural and biophysical parameters control $k_{on}^{\left(mem\right)}$ and when mass action rate constants may not be appropriate to describe membrane-associated binding kinetics.

### Effects of volumetric on-rate, 2D diffusion rate, surface density and structural features on dimerization kinetics

**Figure 2.**
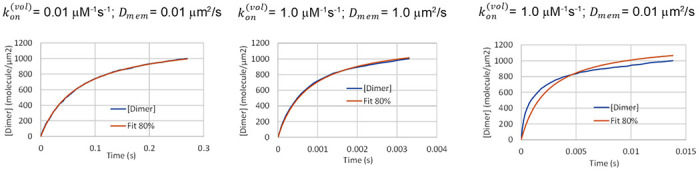
Examples of SpringSaLaD simulation data and their fit to surface-confined mass action dimerization kinetics. The initial surface density of monomers in each case is 2500 molecule/μm^2^. The diffusion coefficient of the anchor and the volumetric binding rate constant of the binding sites are indicated above each graph, corresponding respectively to rows 1, 6 and 5 of Table 1. Each of the SpringSaLaD simulations were run to 80% completion.

We start by analyzing the case of a 5nm stiff tether between a 1nm diameter binding sphere and the membrane. The diffusion coefficient for the binder sphere is set to 1μm^2^/s. Table 1 gives results for all combinations of two volumetric on rate constants, two surface densities and two membrane diffusion coefficients. All the dimerization rate laws are irreversible except for the last 2 rows, where the off-rate constant is indicated. The 2D on-rate constant derived by fitting the SpringSaLaD simulation, $k_{on}^{\left(mem\right)}$, is in the 5^th^ column; for consistency, all these fits are performed for kinetics at 80% completion. For comparison we also provide the 2D on-rate constant, $k_{on}^{\left(h\right)}=k_{on}^{\left(vol\right)}/h$, where *h* is the linker length plus the radius of the binder sphere. If *h* is in units of μm, dividing by a unit conversion factor of 602.2 converts $k_{on}^{\left(h\right)}$ from units of μM^−1^s^−1^ to units of μm^2^molecules^−1^ s^−1^; $k_{on}^{\left(h\right)}$ is an equivalent 2D binding rate constant of freely diffusing monomers confined within a thin layer with a height *h* adjacent to the membrane. The last column provides the ratio of the sum of the squared deviations to the sum of the squared SpringSaLaD mean values; this ratio provides a measure of the goodness of fit to the bimolecular mass action rate law, with anything less than ~10^−3^ representing a good fit. Examples of the fits are shown in Figure 2.

We looked at two anchor diffusion coefficients corresponding to that of a large transmembrane protein domain (*D_mem_*= 0.01 μm^2^/s) and a lipid anchor (*D_mem_*=1 μm^2^/s). The first row of Table 1 considers a case where diffusion of the binder (*D_vol_*=1 μm^2^/s) is 100 times that of the anchor site in the membrane. Because the linker is stiff, the anchor acts as a pivot and the binder rapidly moves within a hemispherical shell to effectively create a reaction region with a thickness of slightly larger than the 1-nm diameter of the binder sphere. Because the region of spatial overlap of the two shells where the binding may occur is restricted, it might seem surprising that $k_{on}^{\left(mem\right)}$ is so closely approximated by $k_{on}^{\left(h\right)}$. However, as shown in the Supporting Information, the reaction probability is enhanced because of the effectively higher density of binding sites within this restricted region, which compensates for the smaller spatial overlap. Thus, the calculations in the Supporting Information both explain and validate the SpringSaLaD results. The second row of Table 1 corresponds to the case where the anchor and the binder have the same fast diffusion. In this scenario, that $k_{on}^{\left(mem\right)}≅k_{on}^{\left(h\right)}$ is intuitive, because the effect of the tether in this case essentially reduces to confining the binders within the layer adjacent to the membrane. The solutions in the Supporting Information assume that the molecular distributions are spatially uniform at any time, which pertains to the cases of the first two rows of Table 1 where $k_{on}^{\left(h\right)}<D_{mem}$.

It has been shown that the rates of membrane reactions may be susceptible to deviations from a simple mass action rate law (13–17), which manifests themselves as significant changes of the apparent rate constant with initial surface density. These changes need to be assessed before using the rate constants we obtain by the procedure of Fig. 1 in large scale cell-level models. We probed for this by decreasing the initial density by a factor of 100. The combinations of *D_mem_*and $k_{on}^{\left(vol\right)}$ in the third and fourth rows of Table 1 resulted in relatively small changes in $k_{on}^{\left(mem\right)}$, indicating that the mass action rate law applies to these cases. We further tested this by increasing the $k_{on}^{\left(vol\right)}$ by a factor of 100 in the lower half of Table 1. Clearly, for the case of *D_mem_*=0.01 μm^2^/s, there is a strong dependence on surface density. Furthermore, this value of *D_mem_* is below the “well-mixed” limit given by $k_{on}^{\left(h\right)}$. Thus, the combination of $k_{on}^{\left(vol\right)}=1.0\;\mu M^{-1}s^{-1}$ and *D_mem_* = 0.01 μm^2^/s present cases where mass action rate laws would not apply. In general, it would not be appropriate to use a mass action rate law, if the apparent $k_{on}^{\left(mem\right)}$ is significantly greater than *D_mem_*. Of course, the appropriateness of the mass action rate law can also be judged by the goodness of fit when determining $k_{on}^{\left(mem\right)}$ from the SpringSaLaD simulation (last column of Table 1); as demonstrated in Fig. 2, the case of $k_{on}^{\left(vol\right)}=1.0\;\mu M^{-1}s^{-1}$ and *D_mem_*= 0.01 μm^2^/s is not fit very well to a mass-action dimerization rate. The simulation results are initially faster and then ultimately slower than the best fit that assumes mass action kinetics. This is because the monomers whose binding sites are initially close to each other (effectively within the “reach” of the tether) will react, but leave behind depletion zones where monomers are too far away from potential binding partners (15, 16). These isolated monomers can be discerned toward the end of Movie 1, which presents an example trajectory for this case.

Consistent with the idea of depletion zones, the last 2 rows of Table 1 show that when reversibility is introduced, the fitted on-rate constant increases (compare, respectively, to rows 5 and 7 of Table 1). This is because when dimerization is reversible, free monomers can reappear to fill in depletion zones, thereby countering the slow diffusion. While the fits to mass action kinetics are still poor, especially for the low density case, the respective mean steady-state densities of dimers in the SpringSaLaD simulations, 570 and 5.7 molecules/μm^2^, are consistent with the thermodynamic law of mass action (18). According to this law, the ratio of the squared monomer volumetric concentration and the dimer volumetric concentration is determined at steady state by the dissociation constant, $K_{d}=k_{off}/k_{on}^{\left(h\right)}$.

Table 2 provides results for three computational experiments in which structural features of the SpringSaLaD molecules are varied. These are all for dimerization reactions where the maximum distance between the membrane and the binding site is 4 times longer than in Table 1: *h* = 0.0205 μm (linker length of 20nm and binding site radius of 0.5nm). The $k_{on}^{\left(h\right)}$ is therefore a factor of 4 slower than that in Table 1 for the same $k_{on}^{\left(vol\right)}$ of 1μM^−1^s^−1^. The SpringSaLaD simulations were carried out with a slow membrane-anchor diffusion coefficient, *D_mem_*=0.01μm^2^/s, to model a membrane receptor. The first row can be directly compared to the fifth row of Table 1, where the only difference is the length of the linker. As would be expected from the increase in *h*, the membrane on rate constant is decreased; however, importantly, this constant is now closer to the diffusion rate and therefore deviations from mass action are significantly reduced. The second row in Table 2 shows results for a structure where additional spherical sites are introduced between the membrane anchor and the binding site to model the space occupied by a cytosolic protein sequence; this steric effect results in a small decrease in $k_{on}^{\left(mem\right)}$. This decrease is reversed when flexibility is introduced by allowing the spherical sites to be pivot points in the third row of the Table; this is how disordered domains may be modeled in SpringSaLaD. Overall, for these idealized structures and simple dimerization, the fitted on-rate constants in Table 2 are relatively close to $k_{on}^{\left(h\right)}$.

### Application of the method to interaction of receptor-bound SOS with Ras.

Till now, we have employed idealized molecular structures to validate our method and to learn some biophysical principles that control the on-rate constants of binding sites tethered to membranes. We now illustrate the application of this approach to a biologically relevant example, namely the interaction of the G-protein exchange factor (GEF) SOS with the lipid anchored small G-protein Ras (19).

SOS has 2 binding sites for Ras: an allosteric site and a catalytic site. When a Ras molecule binds to the allosteric site it increases the GEF activity of the catalytic site (20). Additionally, before SOS binds to Ras it is first recruited to an active receptor tyrosine kinase (RTK) through an adapter protein; the adaptor binds to a proline rich motif (PRM) on SOS via a SH3 domain and to a phosphorylated tyrosine via a SH2 domain. One such RTK is the Epidermal Growth Factor Receptor (EGFR) and one such adaptor protein is Grb2 (21). Once SOS is bound to Grb2, it becomes membrane tethered and its interaction with Ras is facilitated (20). However the complex mechanistic details are still emerging (22).

**Figure 3.**
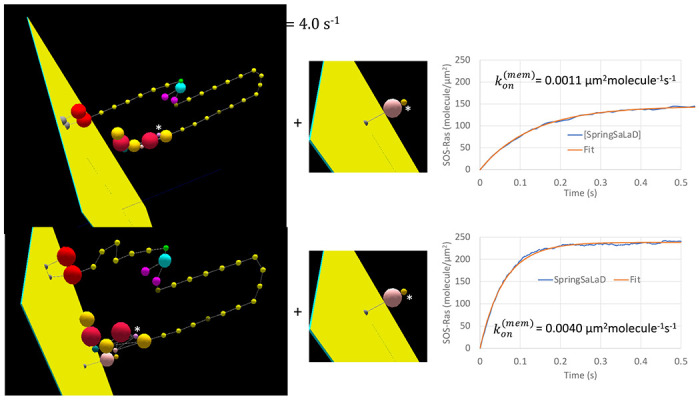
Membrane binding of Ras to the catalytic site of Receptor-bound SOS. Top: direct binding. Bottom: after pre-binding of Ras to the allosteric site. The molecular structures are approximated by using the SpringSaLaD 3D editing utility based on atomic structures derived from AlphaFold2. The top left structure is an EGFR cytoplasmic domain anchored to the membrane (red kinase domain, followed by yellow disordered tail capped by a phosphotyrosine in green); the latter is linked to a cyan SH2 domain in Grb2; one of its magenta SH3 domains is linked to an olive PRM on the end of the disordered region of SOS; the violet SOS binding site for the catalytic domain of Ras is indicated with an asterisk (*). The bottom left structure is identical, except that the pink allosteric site on SOS is bound to Ras. The Ras structures are shown in the center with the yellow binding sites indicated by an asterisk. The input rate constants for the SpringSaLaD simulations are shown at the top, corresponding to the volumetric on rate for Ras binding to the catalytic site of SOS. The EGFR anchor diffusion coefficient is 0.01μm^2^/s. All other site diffusion coefficients are 1.0μm^2^/s. For each condition, 20 EGFR-Grb2-SOS molecules react with 200 Ras molecules on a 250nmX250nm membrane surface to generate 100 SpringSaLaD trajectories. Their means were fit to a deterministic 2D rate law to derive $k_{on}^{\left(mem\right)}$, fixing *k_off_* at 4.0 s^−1^; results for the 2 conditions are shown on the right.

We asked the limited question of how binding of Ras with the receptor-associated SOS catalytic site might depend on whether SOS is prebound to Ras at the allosteric site. Ras is a lipid-anchored protein, so we reasoned that binding of Ras to the allosteric site of SOS would bring the SOS catalytic site closer to the membrane to enhance binding to a second Ras and subsequent exchange of GDP for GTP. Just how big an effect this is, may be estimated by the procedure developed above, with the results shown in Fig. 3.

We developed molecular models with the aid of the mol2sphere (23) utility within SpringSaLaD and were guided by AlphaFold 2 atomic structure predictions (24, 25); all the site diameters and linker lengths are available in the SpringSaLaD input file included in the supporting information; snapshots of the structure are shown in Fig. 3. The top of Fig. 3 displays results for binding of SOS-Grb2-EGFR to Ras at the SOS catalytic site; the bottom shows results for the same reaction, except SOS-Grb2-EGFR had been first bound to a Ras molecule at the SOS allosteric site. The on and off rates shown at the top of Fig. 3 (19) are applied to both of the reactions. In these models, the EGFR membrane anchor site is assigned a diffusion coefficient of 0.01 μm^2^/s to represent a large transmembrane protein, while the Ras membrane anchor is assigned a diffusion coefficient of 1.0 μm^2^/s to represent a lipid anchor; all the sites that are dangling in the cytosol volume are given *D_vol_* of 1.0 μm^2^/s, but since the binding reaction is not close to diffusion-limited, the precise values are not critical. Consistent with these being reaction-limited on-rates, the SpringSaLaD simulation outputs (averages of 40 runs) are well fitted to reversible mass action kinetic law, as shown in the plots on the right of Fig. 3 (relative squared deviations are, respectively, 4.1 X 10^−4^ and 2.0 X 10^−4^). Importantly, the 2D on-rate constants $\left(k_{on}^{\left(mem\right)}\right)$ derived from these fits are, respectively, 1.1 X 10^−3^ μm^2^molecules^−1^s^−1^ and 4.0 X 10^−3^ μm^2^molecules^−1^s^−1^. Likewise, the affinity of the catalytic is site is increased by allosteric site pre-association: *K_d_* = 3600 molecules/μm^2^ for the top of Fig. 3 and 1000 molecules/μm^2^ for the bottom pre-association case. Thus, SOS allosteric site association with Ras is estimated to accelerate its catalytic site binding and affinity by a factor of ~4 – even when SOS is already confined to the membrane through Grb2-mediated association with EGFR.

## Discussion

The kinetics of reactions at membranes have long fascinated biophysicists (1, 3–5, 8, 10, 14, 17). These studies have produced theoretical insights to illuminate how surface-associated reactions have distinct properties compared with reactions occurring in 3D solution. Which of these special properties are most pertinent to any given membrane-bound molecular interaction is difficult to ascertain *a priori*. Furthermore, experiments to measure bimolecular kinetics on membrane surfaces are difficult, so often only on-rate constants measured in 3D are accessible. Fundamentally, however, the kinetics of key membrane-associated reactions depend on the 2D surface densities and 2D rate constants, not on the bulk cellular concentrations and 3D rate constants. Indeed, because surface to volume ratios of different cell types vary tremendously, volumetric rate constants cannot be readily used to model and simulate cell signaling systems. To address these theoretical and practical problems, we describe a procedure (Fig. 1) using experimentally accessible volumetric on-rate constants, $k_{on}^{\left(vol\right)}$ with the SpringSaLaD simulation software to estimate the $k_{on}^{\left(mem\right)}$, the 2-dimensional rate constant for a membrane-confined bimolecular reaction.

To validate the method, we applied it to the dimerization of a single binding site tethered to a surface through a 5nm stiff linker, where the membrane anchor acts as a pivot (Table 1). For the situation where the reaction is rate limiting, this system can be solved analytically (see Supporting Information); gratifyingly, $k_{on}^{\left(mem\right)}$ determined by our method is well reproduced by the analytical solution. Interestingly, for these cases, $k_{on}^{\left(mem\right)}$ is well approximated by $k_{on}^{\left(h\right)}=k_{on}^{\left(vol\right)}/h/602.2$ (μm^2^molecules^−1^s^−1^), where *h* is the distance of the binding site from the membrane anchor. This parameter has also been referred to as the “confinement length” (8), defining a thin volume above the membrane that concentrates the binding sites and directly producing the relationship between $k_{on}^{\left(h\right)}$ and $k_{on}^{\left(vol\right)}$.

While the mass action kinetics are generally applicable for both encounter-limited and reaction-limited kinetics in 3D solution (but see (15)), it has long been appreciated that the situation may be more complex for 2D kinetics (3, 5, 14, 17, 26). This is demonstrated by the results in Table 1 for situations where the diffusion coefficient of the anchor is slow, but the volumetric on-rate constant is fast. For these cases, different estimates of $k_{on}^{\left(mem\right)}$ are obtained at different initial surface density – clearly incompatible with the mass action kinetics. Indeed, the third panel of Figure 2 shows that the SpringSaLaD kinetic data is not well fitted by a 2D mass action rate law. A video of one of these trajectories (Movie 1) nicely illustrates how the initial rate is fast, while the binding sites are within “reach” (10), but falls off as binding sites are left orphaned outside the reach of the remaining slowly diffusing monomers. The results allow us to generalize that mass action applies as long as *D_mem_* is close to or greater than $k_{on}^{\left(h\right)}$. Furthermore, a mass action rate law applies well to the initial rate, before depletion zones develop. Thus, our analysis provides an approach to determine whether 2D binding might be well approximated by the mass action kinetics, and if so, to estimate the 2D mass action on-rate constant.

To explore how other molecular structural features might affect dimerization of the monomers tethered to the membrane, we looked at 3 additional idealized systems in Table 2. In all these, *h* was 20.5nm (as opposed to 5.5nm in Table 1). As expected, the longer confinement length decreased the estimated $k_{on}^{\left(mem\right)}$ by a factor of about 4. The insertion of steric sites between the anchor and the binding site or allowing for flexibility of the linker region have minor effects on $k_{on}^{\left(mem\right)}$, which is relatively well approximated by $k_{on}^{\left(h\right)}$. Importantly, the lower value of $k_{on}^{\left(h\right)}$ for this system was closer to *D_mem_*, resulting in a better fit by the mass action kinetics than for the similar case with the 5nm linker (5^th^ row of Table 1).

For membrane binding of real biological molecules, it may be difficult to estimate the average location of a binding sites relative to the membrane surface (i.e. *h*); also, the 2 binding sites may be parts of very different structures with different distances from the membrane surface. In situations like this, our approach has the potential to provide good estimates of rate constants that can be applied to larger cell signaling systems. Indeed, there may be direct insights that can be realized just by considering the structural details of the interacting membrane molecules. We have illustrated this in relation to adaptor-mediated protein kinase receptor signaling mechanisms, specifically for the interaction of the GEF SOS with its effector Ras (Fig. 3). It has been shown that direct catalysis by the SOS catalytic domain of Ras conversion from the GDP to the GTP states is relatively slow. However, prebinding binding of Ras to SOS at a site that is not catalytic (termed the “allosteric” site on SOS) significantly accelerates the catalytic activity, where the catalysis becomes processive (19, 20, 22). The results in Fig. 3 suggest that at least part of this acceleration may be due to the close proximity of the SOS catalytic site to the membrane once it is bound to Ras at its allosteric site. Even though SOS is already localized to the membrane by initially binding to EGFR via Grb2 in our computational experiment, pre-binding of the SOS allosteric site to Ras brings it to still closer proximity to the membrane. Of course, there could be additional effects such as a direct allosteric enhancement through a conformational change or release of self-inhibition (19, 20, 22), but here we focus on the significance of constricting the binding zone through membrane tethers of varying length and flexibility. Our approach toward deriving membrane on-rates will aid in the parametrization of ODE and PDE models that could help elucidate the full kinetic and mechanistic details of processes such as Ras activation and downstream signaling.

## Methods

All simulations were performed with SpringSaLaD v. 2.3.4 (https://vcell.org/ssalad). To build coarse grained molecular structures used in SpringSaLaD for Fig. 3, we used AlphaFold2 (24, 25) to generate PDB file estimates of protein structures for EGFR, Grb2, SOS, and Ras via input of entire amino acid sequences. These PDB files are converted to coarse-grained molecular models via the mol2sphere (23) utility embedded in SpringSaLaD. In some cases, we manually edited the structures to capture their essential features from measurements on the PDB structures, as visualized in PyMol (Schrödinger, Inc.). In particular, for SOS we subdivide the CDC25 and REM domains into multiple smaller spherical sites with only one site capable of participating in a binding reaction. This process maintains the structural characteristics of these domains, while ensuring that the binding radius of the domain is not artificially inflated. We also consider molecule flexibility when making user modifications to molecule structure. When PDB files are imported to SpringSaLaD via mol2sphere, the default is for each site to be linked to no more than 2 immediately adjacent sites. Flexibility can be decreased by introducing more stiff linkers to more adjacent spherical sites. We employ this method to ensure that our multi-site representation of CDC25 and REM domains diffuse as a fixed group of spherical sites instead of individual, highly flexible domains. Modeling disordered regions, such as the PRM region of SOS, can be challenging due to low confidence in the AlphaFold2-generated geometry of these regions. To model disordered domains, we use PyMOL to measure the length of entire straight chain amino acid sequences, then model this sequence in SpringSaLaD using 1.0 nm diameter sites connected by 3.1 nm linkers. Binding reactions in all simulations have rates input in terms of μM^−1^s^−1^; successful binding results in 1nm (Tables 1 and 2) or 0.5 nm (Fig. 3) distances between the surfaces of the spherical sites. A set of 100 trajectories are simulated in parallel using the Center for Cell Analysis and Modeling High Performance Compute Cluster (https://health.uconn.edu/high-performance-computing/resources/). SpringSaLaD input files are in Supporting Information and provide all the geometric details for the molecules in each computational experiment.

The mean of 100 SpringSaLaD simulations for each of the simulations in Tables 1 and 2 and Fig. 3 were fit to a deterministic 2D binding rate law to obtain $k_{on}^{\left(mem\right)}$. For irrreversible dimerization (Table 1, first 8 rows and Table 2), a fit to an analytical expression (Eq. 1) used the Excel solver; for reversible dimerization (Table 1, rows 9 and 10) and for the fits in Fig. 3, we used the COPASI (27) parameter estimation tool within Virtual Cell (VCell) (28, 29). The latter can be accessed in the VCell published BioModel “Peterson Figure 3: Ras-SOS_Binding_fit_to_SpringSaLaD”. All these results with some further analysis can also be found in the spreadsheets included in the Supporting Information.

## Supplementary Material

1

## Figures and Tables

**Table 1.** Results for single stiff link, binding site *D_vol_*=1.0μm^2^/s, *h*=0.0055μm: 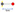

$k_{on}^{\left(vol\right)}$ (μM^−1^s^−1^)	Surface density (molecules/μm^2^)	*D_mem_* (μm^2^/s)	$k_{on}^{\left(h\right)}=k_{on}^{\left(vol\right)}/h/602.2$ (μm^2^*molecules^−1^*s^−1^)	$k_{on}^{\left(mem\right)}$, SpringSaLaD fit (μm^2^*molecules^−1^*s^−1^)	Goodness of fit (Relative Sq Dev)
0.01	2500	0.01	3.0X10^−3^	2.9 x 10^−3^	3.0 x 10^−5^
0.01	2500	1.0	3.0X10^−3^	3.1 x 10^−3^	3.1 x 10^−5^
0.01	25	0.01	3.0X10^−3^	2.4 x 10^−3^	2.9 x 10^−5^
0.01	25	1.0	3.0X10^−3^	3.0 x 10^−3^	2.3 x 10^−5^
1.0	2500	0.01	0.30	0.08	7.4 x 10^−3^
1.0	2500	1.0	0.30	0.26	2.6 x 10^−4^
1.0	25	0.01	0.30	0.02	1.5 x 10^−3^
1.0	25	1.0	0.30	0.20	4.9 x 10^−4^
1.0 (*k_off_*=10^3^s^−1^)	2500	0.01	0 30	0.18	1.3 x 10^−3^
1.0 (*k_off_*=10s^−1^)	25	0.01	0.30	0.1	6.2 x 10^−2^

**Table 2.** Dimerization of idealized structure types.

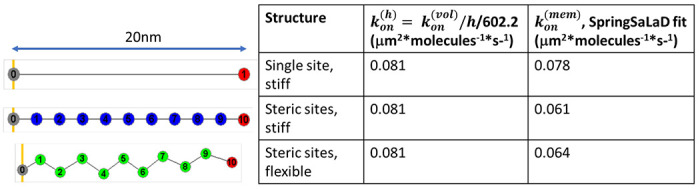

*D_mem_*=0.01μm^2^/s, binding site *D_vol_*=1.0μm^2^/s, $k_{on}^{\left(vol\right)}=1\;{\text{μM}}^{\text{−1}}{\text{s}}^{\text{−1}}$, *h*=0.0205μm, surface density = 2500 molecules/μm^2^

## SIGNIFICANCE STATEMENT.

In cell signaling, the activation of a surface receptor leads to a cascade of intracellular biochemical events. Many of these occur near the inner plasma membrane surface. However, accurate rate parameters for these initial steps in models of signaling are rarely available because membrane-tethered reaction kinetics are difficult to experimentally measure. Here, we use a highly coarse-grained molecular simulator to model the kinetics of reactions between binding sites that are tethered to a membrane. We can fit these simulation outputs to 2-dimensional rate laws to obtain rate constants that can be used to build complex models of cell signaling. These rate constants can also be compared to understand the key biophysical features controlling the kinetics of bimolecular membrane reactions.
