# Supplementary material for: Bridging molecular to cellular scales for models of membrane receptor signaling
Source: bioRxiv. 2024 Dec 5:2024.12.04.626844. Preprint. [Version 1] doi: 10.1101/2024.12.04.626844 (PMC11643039; doi:10.1101/2024.12.04.626844)
Supplement: 1 [file NIHPP2024.12.04.626844v1-supplement-1.pdf]

bioRxiv preprint doi: https://doi.org/10.1101/2024.12.04.626844; this version posted December 5, 2024. The copyright holder for this preprint (which was not certified by peer review) is the author/funder, who has granted bioRxiv a license to display the preprint in perpetuity. It is made available under aCC-BY 4.0 International license.

List of Supporting Data and Information

Derivations for limiting cases related to Table 1. Filename: Supporting Text - Derivations for limiting cases.docx

SpringSaLaD input files for the models in Tables 1 and 2 and Figure 3. Filenames: Table 1 and 2 SIMS.zip; EGFR_Grb_SOS binding to Ras.txt; EGFR_Grb_SOS_prebound at allo binding to Ras.txt

Spreadsheets related to Tables 1 and 2 and Figure 3 containing the SpringSaLaD simulation results and the fits to determine $k^{(\qquad)}$. Filenames: Table 1 5nm_stiff_fits.xlsx; Table2 20nmT_fits.xlsx; Data for Figure 3.xlsx

Movie of an example trajectory for the model of Table 1 Row 5 and also Fig. 2, 3rd panel. Filename: Movie kon=1 Danchor=membrane protein.mp4
